# Different solutions lead to similar life history traits across the great divides of the amniote tree of life

**DOI:** 10.1186/s40709-021-00134-9

**Published:** 2021-02-08

**Authors:** Shai Meiri, Gopal Murali, Anna Zimin, Lior Shak, Yuval Itescu, Gabriel Caetano, Uri Roll

**Affiliations:** 1 School of Zoology, Tel Aviv University, 6997801 Tel Aviv, Israel; 2 The Steinhardt Museum of Natural History, Tel Aviv University, 6997801 Tel Aviv, Israel; 3 Mitrani Department of Desert Ecology, The Jacob Blaustein Institutes for Desert Research, Ben Gurion University of the Negev, Midreshet Ben Gurion, Israel; 4 Leibniz-Institute of Freshwater Ecology and Inland Fisheries (IGB), 12587 Berlin, Germany; 5 Institute of Biology, Freie Universität Berlin, 14195 Berlin, Germany

**Keywords:** Amniotes, Aves, Breeding frequency, Cleidoic egg, Clutch size, Ectothermy, Endothermy, Litter size, Mammalia, Metabolic rates, Offspring size, Parental care, Reproductive investment, Reptilia, Squamata

## Abstract

Amniote vertebrates share a suite of extra-embryonic membranes that distinguish them from anamniotes. Other than that, however, their reproductive characteristics could not be more different. They differ in basic ectothermic vs endothermic physiology, in that two clades evolved powered flight, and one clade evolved a protective shell. In terms of reproductive strategies, some produce eggs and others give birth to live young, at various degrees of development. Crucially, endotherms provide lengthy parental care, including thermal and food provisioning—whereas ectotherms seldom do. These differences could be expected to manifest themselves in major differences between clades in quantitative reproductive traits. We review the reproductive characteristics, and the distributions of brood sizes, breeding frequencies, offspring sizes and their derivatives (yearly fecundity and biomass production rates) of the four major amniote clades (mammals, birds, turtles and squamates), and several major subclades (birds: Palaeognathae, Galloanserae, Neoaves; mammals: Metatheria and Eutheria). While there are differences between these clades in some of these traits, they generally show similar ranges, distribution shapes and central tendencies across birds, placental mammals and squamates. Marsupials and turtles, however, differ in having smaller offspring, a strategy which subsequently influences other traits.

## Introduction

The emergence of amniotic vertebrates was a remarkable event in the evolutionary history of life on Earth. Amniotes developed a triumvirate of extraembryonic membranes: the amnion, the allantois, and the chorion. This had enabled them to successfully colonize the terrestrial environment and eliminate the need to reproduce in water [1]. Arguably these membranes allowed them to evolve a host of morphologies and ecologies and become the dominant form of life on Earth (at least in term of body size, and its associated influence on food webs, vegetation structure etc.).

The evolution of the amniotic developmental strategy was associated with a general enlargement of the embryos, and a general reduction in their numbers, compared to anamniote vertebrates. Some fishes can lay millions of tiny eggs (e.g., the Atlantic cod, *Gadus morhua* [2, 3]; *Sebastes* sp. rockfishes [4]; the common ling, *Molva molva* [5]; the ocean sunfish, *Mola mola* [6]). Some anurans (mostly bufonids) can lay 35,000 or more eggs in a clutch (e.g., the cane toad, *Rhinella marina* [7], the green toad, *Bufotes viridis*, [8], the Great-Plains toad, *Anaxyrus cognatus*, can lay over 45,000 eggs, [9] and *Rhinella jimi* may lay over 50,000 eggs, [10]). Despite being, on average, much larger bodied than amphibians, the maximum clutch size of amniotes is over two orders of magnitude smaller (maximum 242 eggs in the hawksbill sea turtle, *Eretmochelys imbricata* [11]). It is logical to assume that the complexity and energetic cost of the amniote embryonic development sets a much higher bar for minimum offspring size [12] than the relatively simple embryogenesis of anamniotes.

While sharing the basic set of extraembryonic membranes, major amniote clades have diverged from one another, over hundreds of millions of years, adopting widely disparate physiologies, morphologies [1] and reproductive strategies. Active flight constrain the output of a single reproductive event (so as not to impose a burden of the mother carrying eggs or, in bats, neonates; e.g., [13]). It may explain why birds produce one egg per day whereas reptiles lay the entire clutch in minutes or hours, and the degree of parental care in birds was found to be negatively related to productivity rates [14]. Flight evolved independently in three amniote lineages (two of them extant). Endothermy evolved twice in extant amniotes, in the lineages leading to crown group mammals and birds. Endotherms lead much more energetically expensive lives than ectotherms. At rest, endotherms spend ten times the energy than similar-sized ectotherms do [15]. This means that endotherms spend more of their energy on maintenance, whereas ectotherms spend relatively more of their available energy on reproduction (e.g., [16, 17], see also Bonnet et al. [18]). Endotherms therefore spend much more energy to produce a similar biomass of offspring [19]. Some have argued that this allows ectotherms to out-do endotherms in terms of both numbers of offspring and biomass production rates [20, 21].

### Viviparity and parental care

Viviparity evolved once in therians—and dozens of times in squamates [22], but crucially never (as far as we know) in extant archelosaurs (turtles, birds, and crocodiles). Parental care (here: only in the form of post-partum/post-hatching care) also probably evolved once in birds (or in the lineage leading to birds; [23]) and once in mammals [24]. It evolved several times in squamates [25–27], but most species of squamates do not care for their young [28–31]. Crocodiles tend their eggs and young for a short period [26, 28, 30], while turtles do not. Yet, it was the endotherm revolution that became associated with ubiquitous, and often lengthy, parental care [25, 31, 32] which always extends to the post-hatching/birth period. It is associated with extensive parental provisioning of both food and warmth. The reptilian version of parental care, on the other hand, is more rudimentary. It is often limited to the eggs, does not extend to provisioning of the young, and is usually a much simpler, shorter affair where mothers at best tolerate their offspring, providing passive defense [18, 26, 30], though active defense can occur, e.g., [33]. In contrast, actively providing food, thermal shelters, and teaching skills are all hallmarks of endotherm parental care. Endotherms nearly always invest in their offspring for a lengthy period after offspring are born or hatch, which necessitates considerable parental energy investment, whereas reptilian offspring are independent at birth, almost never requiring any further parental investment [25, 34].

The evolution of both viviparity and parental care is energetically costly [35, 36] and forces the mother to forgo future opportunities for reproduction. These traits are therefore often thought to promote shifts towards ‘slower’ life histories and towards the K strategy along the r-K continuum (e.g., [37–39]).

In fishes, amphibians and squamates (i.e. vertebrates in which there is variation with regards to both reproductive mode and parental care—unlike birds and therian mammals) live bearers, and species practicing parental care, have smaller broods ([5, 40–43], but see [44, 45] for squamates and [46] for elasmobranchs). Live bearers and species practicing parental care also have larger offspring or eggs than similar-sized oviparous and non-caring species ([6, 41–43, 46–53], but see [54]). Reproductive frequency is lower in viviparous taxa and in those practicing forms of parental care [28, 40, 44, 45, 55–58]. Finally, viviparous taxa mature later ([44, 45, 59, 60] but see [61]), and live longer ([59] but see [62]). These associations have independently evolved in a variety of anamniote vertebrate clades [63] including elasmobranchs [46, 53], teleosts (e.g., [48, 53, 59]), and amphibians (e.g., [43]), as well as in a suit of invertebrate taxa [63].

Within amniote classes, major clades often fundamentally differ from one another in several key life-history characteristics. In birds, perhaps the most uniform of the three classes (in terms of gross morphology and life history strategy), members of the Palaeognathae and Galloanserae, tend to have highly precocial young, whereas altricial young dominate in the Neoaves [64]. The reptilian clades Testudines and Crocodilia are phylogenetically further apart from other reptiles (Lepidosauria) than they are from Aves (birds) (e.g., [65–67]). The highly restrictive morphology of the turtle shell may restrict egg size [68]. Furthermore, while parental care is ubiquitous in crocodiles, it is totally absent in turtles, and rare in squamates ([28, 30] and see above). Thus, it is reasonable to expect divergent life history evolution across clades. Within squamates the repeated evolution of viviparity was long hypothesized to strongly affect a plethora of life history traits, generally creating a shift toward larger, fewer offspring per reproductive event, as well as less frequent reproduction (because embryos are carried in the uterus until parturition [58, 63, 69–71]; cf. [72]). While some have argued viviparous squamates have larger offspring than oviparous ones [43, 63, 70, 71], Meiri et al. [72] have shown this is not the case. In mammals, oviparity is restricted to monotremes, but placentals and marsupials (all viviparous) take fundamentally different approaches to embryonic development [1] and life history [73]. Marsupials are born at a very early ontogenetic stage, and after a much shorter gestation, than placentals [73, 74]. After birth marsupials spend an extended period in the pouch, weaning at an older age than similar-sized placentals [75].

### Life-history allometry

A factor that is well known to govern the evolution of life histories in all animal taxa is body size [76–78]. In some lineages of anamniote vertebrates offspring size scales positively with adult size (e.g., [6, 48, 52, 53, 79]; see also Figure 4 in [80]). In other anamniote lineages, however, mothers lay very small eggs, regardless of their own size, resulting in a flat body size-egg size relationship (e.g., teleosts: [6, 80, 81] cf. [82] and Amphibians: [83, 84]). In amniotes, a positive linear relationship between offspring (or egg) size and adult size across species is ubiquitous (e.g., [57, 75, 85–89] and see below).

The allometry of clutch and litter sizes, however, differs greatly between the amniote classes (e.g., [90] and see below). Clutch sizes generally increase with body size across the animal kingdom, both in invertebrates (e.g., [86, 91–93]) and in anamniote vertebrates (fishes: [53, 80, 82, 94]; amphibians: [6, 40, 52, 79, 84]). Reptile clutch sizes similarly increase with maternal body size [44, 45, 57, 87, 95–97]. Several species-rich lizard lineages (e.g., Gekkota, Gymnophthalmidae, Dactyloidae), however, evolved fixed clutches of one or two eggs, irrespective of body size [12, 98, 99].

In contrast, large sized mammals usually have small litters (most often of a single neonate) while smaller mammals have larger litters ([100–102]; cf. [103]). One major mammalian lineage, however, the Chiroptera (bats), is characterized by invariantly small litters of one or, rarely, two neonates (very rarely more [104]). Likewise the Primates also have litters of only one or two young (e.g., [105, 106]), but even in primates larger species tend to have a litter size of one, and smaller ones bear two offspring [106].

In birds, the relationship between clutch size and body size is usually thought to be negative, similar to the mammalian pattern, but the slope of this relationship is usually very shallow ([77, 101, 107, 108]; cf. [109]). Unlike mammals, however, the largest birds (i.e. the ratites), do not have small clutches (the smallest of them, the Kiwis, *Apteryx* spp. have clutch sizes of one [110] whereas rheas and ostriches have larger clutches). Furthermore, almost all the (invariably large bodied) members of the Galloanserae have large clutches (data from [107]). Clutch size/body size relationships may also differ between altricial and precocial birds [111], cf. [108]. These disparate relationships may result in differences in the allometry fecundity rates (young hatching/born per year) differing across amniote taxa.

Reproduction frequency decreases with body size in all major amniote taxa [57, 87, 101, 112–117]. On the other hand, rates of biomass production [57, 102, 116, 118], age at maturity [59, 61, 76, 87, 114, 119], and longevity [62, 120–123], all increase with body size.

While we envision class-level and other such higher-order differences between the life history characteristics of major amniote taxa, all the quantitative measures we examine also exhibit considerable intra-clade variability. Much of this variation has been attributed to the major disparity in body size within amniote classes ranging across five orders of magnitude in extant birds, seven orders of magnitude in extant reptiles (nine if the extinct mosasaurs are considered) and eight orders of magnitude in mammals (see below).

In reptiles parental care is too rare, and potentially too rudimentary, to explain much of the intra-class variance. Viviparity, however, evolved many times in squamate reptiles [22], and furthermore manifests great variability in the degree of fetal provisioning and placentation—from ovoviviparity to placental mammal-like nutrient exchange [124]. This allows meaningful testing of the effects of viviparity in a phylogenetic context [45, 57, 97, 118, 125–129] which makes the Squamata a fantastic model in which to directly test for the evolutionary implications the evolution of viviparity may have had for life history evolution.

Our working hypothesis is that the major differences in phylogeny, reproductive mode, parental provisioning and thermal biology, will lead to measurable quantitative differences among several reproductive indices. Specifically we hypothesize that: (1) Endothermy (coupled with parental provisioning of birds and mammals) will result in larger offspring than those of similar-sized ectotherms. Thus mammals and birds will have larger neonates and hatchlings than reptiles with similar-sized mothers (cf. [54]); (2) Endotherms will have fewer offspring per litter/clutch than ectotherms, and viviparous squamates will have smaller broods than oviparous ones, because of their higher investment in parental care, and due to constraints imposed by maternal body volume and resource availability necessitate a tradeoff; (3) Squamate viviparity will be associated with less frequent reproduction because of the need to carry embryos to full term in the reproductive tract. Oviparous species on the other hand can lay eggs when embryos are at an early developmental stage and are immediately free to reproduce again. Furthermore, viviparous squamates predominate in high-latitude, highly seasonal, regions [130]—where seasons are short and hence also likely to reproduce less frequently. Because of their lengthy parental care, we predict that mammals and birds reproduce less often than oviparous reptiles; (4) Endothermy and viviparity will therefore be associated with lower rates of biomass production. Alternatively, endothermy accelerates metabolic rate and hence embryonic development may be faster, resulting in accelerated production rates. We test these hypotheses by comparing the life history characters of mammals, reptiles, and birds—with emphasis on the overall variation in these traits rather than on strictly quantitative measures of central tendency.

## Methods

### Data

We obtained data on vertebrate life history and reproduction characteristics from the literature. Data on mammal life histories are from the PanTHERIA dataset [104]. Litter sizes smaller than one in PanTHERIA were changed to one. Data on birds are from Jetz et al. (clutch sizes; [107]), Sibly et al. (annual mass production; [117]), and Myrvhold et al. (all other data; [131]). Data for turtles are from the primary literature. Data for squamates are from Feldman et al. (snake reproductive mode; [130]), Feldman (snake life history; [132]), Meiri et al. (lizard clutch sizes; [97]), and Meiri (all other data for lizards, [54, 133]), supplemented with data from the primary literature for snakes. Data for offspring sizes are from Meiri [54], with minor updates for reptiles.

We collected the following types of data, that are comparable across all taxa: (1) Mean body mass of adult females or (when unavailable) of unsexed adults (in g). (2) Mean body mass of hatchlings or neonates (in g). (3) Clutch or litter size. (4) The number of yearly clutches or litters (henceforth “broods”). Also, the following composite measures were considered: (5) Relative brood mass (i.e. attribute #3 times attribute #4 divided by attribute #2). (6) The number of young produced per year (attribute #4 times attribute #5, individuals × year^−1^). We omitted incorrect values of > 100 offspring a year for two cuckoo and six megapode species [131]. These are more than twice the next value, and we suspect confound clutch size and clutch frequency (e.g., incorrect value of 12 clutches of 9.2 eggs for *Cuculus canorus* cf. [134–136]; erroneous 21 clutches of 14.8 eggs for *Alectura lathami* which actually lay one egg at a time: [137], San Diego Zoo 2018). (7) The biomass produced by a female per year (attribute #3 × attribute #4 × attribute #5, g × year^−1^).

**Fig. 1:**
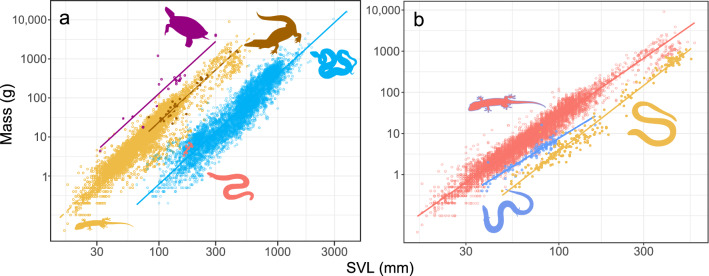
The lengths and weights of specimens at the Steinhardt Museum of Natural History, Tel Aviv University **a** reptiles: 34 crocodiles (brown), 22 turtles (purple), 5110 snakes (blue), 7478 lizard (yellow) and 6 amphisbaenian (red) **b** only for lizards: colored according to leg development: fully developed (red), limb reduced (including two-limbed species; blue) and limbless species (yellow)

Body size data are reported for endotherms as body masses (e.g., [114, 117]). For reptiles, however, mass data are rarely reported [138, 139]. Therefore—and to make sure data are comparable between reptiles and endotherms—we converted lengths to masses using the allometric equations published by Feldman et al. [140]. We note that such conversion is crucial not only for inter-class comparisons but also between squamate clades that differ much in their shape. For example, at the collections of the Steinhardt Museum of Natural History, Tel Aviv University, a 200 mm long snake (*Letheobia simoni*, snout-vent length = SVL, specimen #6397) weighs 0.7 g whereas a dragon lizard, *Pogona barbata* (specimen #14010) of exactly the same SVL (200 mm) weighs over 400 times as much (292 g). A somewhat shorter turtle (*Mauremys rivulata*, specimen #9652, 150 mm carapace length) is even heavier (372 g). There are 12,659 specimens with both mass and either SVL or carapace length data in this collection (including six amphisbaenian, 34 crocodile, 22 turtle, 5110 snake, and 7478 lizard specimens). The OLS slopes of log_10_ mass on log_10_ length regression are similar across taxa (2.79 for turtle carapace length, and for SVLs: 2.80 for crocodiles, 2.87 for lizards and 2.77 for snakes, R^2^ values: 0.82, 0.91, 0.91 and 0.86, respectively). The intercepts, however, are vastly different (− 3.47 for turtles, − 4.22 for crocodiles, − 4.41 for lizards and − 5.72 for snakes). Thus at 150 mm SVL these equations predict a turtle will weigh 403 g, while a (very small) crocodile will weigh 75 g, a lizard 69 g and a snake only 2 g (Fig. 1). Reptiles with similar lengths can be very different in body size. Thus, when comparing sizes across taxa weight is a much more reasonable measure, which is why we converted all lengths to masses.

We define *brood size* as the average number of eggs in a clutch or neonates in a litter. Sizes of neonates and hatchlings are masses (in g) reported at birth or hatching, or lengths converted to masses for squamates, as described above. Adult sizes are mean female masses. We record the mean number of clutches weighed per year.

For birds and mammals we obtained life history data from the large compilations reported above. For reptiles, however, we compiled the data ourselves (see e.g., [133, 141]). In many cases we had multiple data points per species per trait. In these cases, we averaged the smallest and largest reported means. When means were unavailable, we averaged the smallest and largest reported observation (for body size these are minimum and maximum sizes of adults).

We followed the August 2020 taxonomy of Reptile database for reptiles [142], the July 2020 taxonomy of IOC Bird List for birds [143] and the September 2020 taxonomy of Mammal Diversity Database for mammals [144]. We used the ‘taxize’ R package [145], and the Integrated Taxonomic Information System (ITIS 2020) to help resolve synonyms. For phylogenetic analysis, we followed the taxonomy of the phylogenies used.

### Statistical analyses

Because endothermy and obligatory parental provisioning (as well as flight) evolved only twice during the evolution of extant amniotes (in the common ancestors of mammals and birds, and flight in bats and birds) we are unable to meaningfully use phylogenetic comparisons for these traits. Such unique evolutionary events do not lend themselves to methods such as phylogenetic least square regression, as the number of degrees of freedom is effectively nil ([146], see also [147]). The inability to conduct meaningful phylogenetic comparisons of the impact of endothermy and parental care on life history strategies across amniote classes compelled us to use descriptive statistics and graphs to examine differences and similarities between them. To compare allometries between major tetrapod clades we used phylogenetic generalized least square regression (PGLS), implemented in the R package CAPER [148]. In all PGLS analyses we estimated the strength of the phylogenetic signal using the maximum-likelihood parameter λ and used this value to scale branch lengths [148]. To reduce heteroscedasticity and normalize residual distributions, all quantitative data (clutch and litter size, hatchling, neonate and adult female mass, and brood frequencies) were log_10_ transformed prior to analyses. In a couple of occasions [brood size allometry in mammals (Fig. 6) and allometry of relative brood mass (Fig. 10)], we depict the slopes using a Generalized Additive model instead of PGLS as visual examination of scatter plot revealed a non-linear relationship. We used taxonomy-imputed trees from Upham et al. [149] for Mammalia, Tonini et al. [150] for Squamata, Colston et al. [151] for Testudines, and Cooney et al. [152] for Aves to control for phylogenetic autocorrelation. We averaged the life history data of species which are represented by a single synonym in the phylogenetic tree and multiple synonyms in the life history data sets. We compared the phylogenetic signal, slopes, and intercepts of each trait’s regression line between groups, as well as how much variation in each trait is explained by body size.

## Results

### Comparisons of mammals, birds, and reptiles

#### Body size

Amniotes come in many different sizes, but class-level differences are readily apparent. Turtles are distinctively the largest (even the smallest species is larger than the median for all other clades), followed by mammals (which have the highest masses) then birds, and squamates are the smallest. The median-sized mammal, at 79.7 g, weighs more than twice as much as a median size bird (36.4 g), and over 5 times the median size reptile (14.3 g; Table 1)—despite the fact that our estimates for reptiles are based on maximum sizes ([140, 153], Additional file 1: Appendix S1) whereas those for mammals and birds are based on means. Thus, reptile sizes here are inflated by about a factor of about two (maximum species mass is, on average, 1.97 times mean female mass, over 4944 species of lizards; Meiri, unpublished).

**Table 1:** Body masses of amniotes (g)

	n	Mean	Median	Minimum	Maximum	Orders of magnitude
Aves	9281	51	36.4	1.9	109,250	4.8
Mammalia	5364	197	79.7	1.6	190,000,000	8.1
Squamata	10,825	18.2	14.3	0.07	345,144	6.7
Testudines	308	397	2804	93.1	950,000	4

n = number of species with data. Means are back transformed from logarithms

**Fig. 2:**
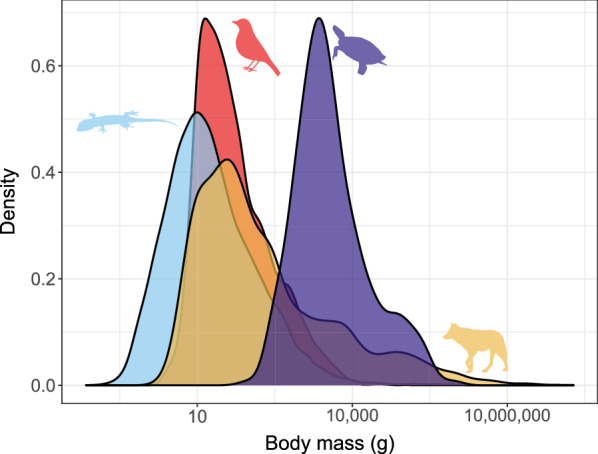
Body mass of amniotes. Masses (in grams) are maxima for ectotherms and means for endotherms. Red: birds, blue: squamates, purple: turtles and yellow: mammals (both placentals and marsupials). Note that the x axis is log_10_ scaled

The distributions of sizes within the three classes have similar shapes, all are unimodal and right-skewed (Fig. 2). That said, reptiles attain much smaller sizes than endotherms, and dominate size classes up to ~ 10 g, mammals attain the largest sizes, and dominate size classes > 1 kg, whereas bird sizes are intermediate and less variable, and 67% of bird species occupy a size range between 10 and 180 g (Fig. 2).

#### Offspring size

**Fig. 3:**
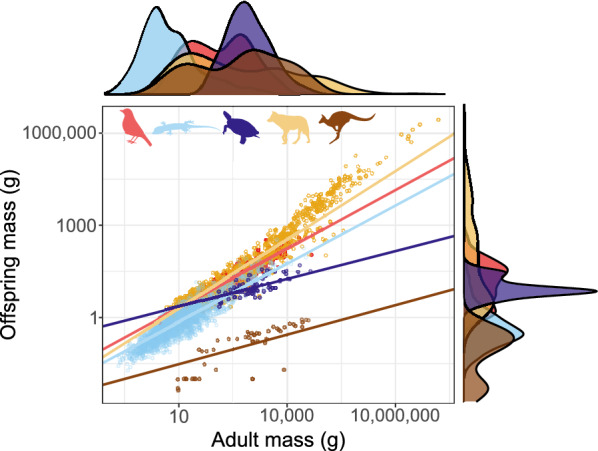
Allometry of offspring size. Red: birds; blue: squamates; purple: turtles; yellow: placental mammals; brown: marsupial mammals. Solid lines are regression slopes from PGLS analysis. Both axes are log_10_ scaled

**Fig. 4:**
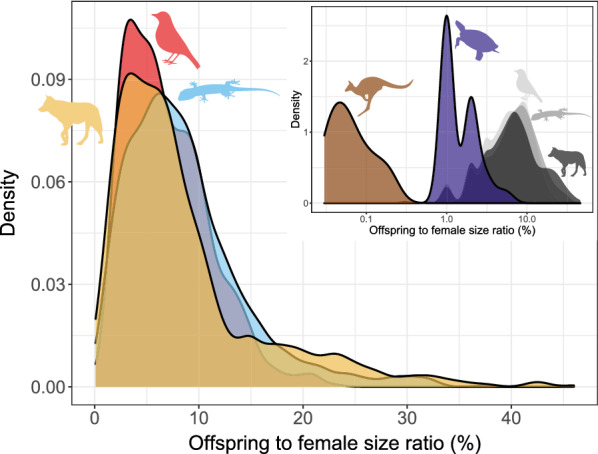
Offspring size (expressed as % of offspring size to the size of adult females). Red: birds; blue: squamates; purple: turtles; yellow: placental mammals; brown: marsupial mammals. Note that the x axis is log_10_ scaled in the inset figure. Size ratio close to zero (< 0.03) only occur in marsupials (inset; values < 0.03 not shown)

Offspring mass increases with female mass across all clades (squamates: slope 0.631, intercept − 0.748, R^2^ = 0.649, n = 2073, *p* < 0.001, λ = 0.824; birds: slope = 0.637, intercept = − 0.353, R^2^ = 0.768, n = 716, *p* < 0.001, λ = 0.955; placental mammals: slope 0.749, intercept = − 0.477, R^2^ = 0.771, n = 1012, *p* < 0.001, λ = 0.952; Fig. 3) (see [154] for a discussion of the nuances of the interpretation of R^2^ in PGLS). That said, the allometric slopes for turtles (slope: 0.301, intercept 0.049, n = 75, *p* < 0.001, λ = 0.879) and marsupials (Metatheria; slope: 0.323, intercept − 1.850, n = 52, *p* < 0.001, λ = 1) are decidedly shallower, and the relationship weaker (R^2^ = 0.498 for turtles, R^2^ = 0.438 for marsupials). The offspring/female size ratio is very similar across the three major groups: 8.2%, on average, in both mammals and squamates, and 7.0% in birds, and shows remarkably similar distributions (Fig. 4). The mammal data hides great disparity between placentals (mean 8.6%) and marsupials (mean 0.04%). Turtles further deviate from the overall pattern having relatively small young (on average 1.3% of the mother size).

#### Brood (clutch or litter) size

**Fig. 5:**
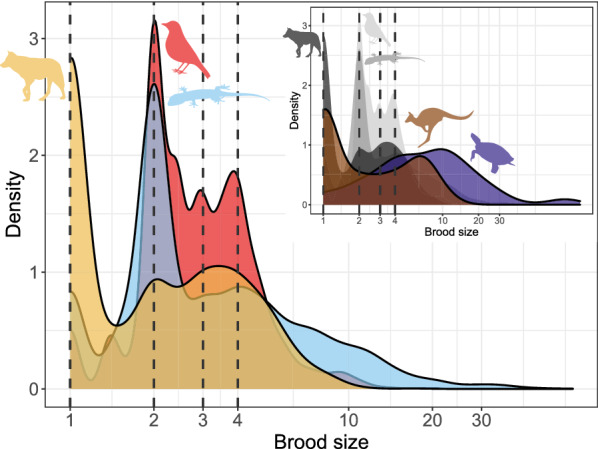
Brood size. Red: birds; blue: squamates; purple: turtles; yellow: placental mammals; brown: marsupial mammals. Dashed vertical lines represent brood sizes of 1 (left-end), 2, 3 and 4. Note that the x axis is in log_10_ scale

**Fig. 6:**
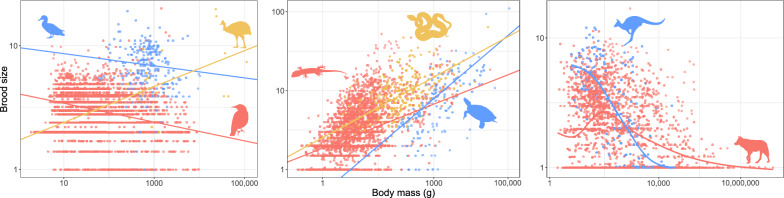
Allometry of brood size. From left to right; **a** Aves (red: Neoaves, blue: Galloanserae, yellow: Palaeognathae), **b** Reptilia (red: Sauria, blue: Testudines, yellow: Serpentes) and **c** Mammalia (red: Eutheria; blue: Metatheria). Solid lines are regression slopes from PGLS analysis for Aves and Squamates. For Mammalia, regression lines are from a Generalized Additive model with knots (k = 10)

Brood sizes are higher in squamates (mean 4.960; n = 5131 species) than in birds (3.1, n = 5297) and mammals have the smallest broods (2.5, n = 2500). That said, the shape of the brood size distribution is similar overall (Fig. 5), with the mean distinction being that the mammalian mode is one neonate, whereas both birds and squamates have a mode of two hatchlings. From these modes the frequencies decrease as brood sizes increase in all three clades. Turtles are a clear exception, however, with very large clutches (12.1 eggs on average, n = 294 species)—a value only reached by 7.5% of squamates, 0.2% of birds, and three mammal species (*Tenrec ecaudatus, Gracilinanus agilis,* and *Monodelphis dimidiata*). However, major differences in reproductive allometry are apparent within the major above clades: in birds the relationship between (log_10_) clutch size and (log_10_) body mass is nearly flat in all taxa (Galloanserae: slope: − 0.035, R^2^ = 0.002, n = 288, *p* = 0.446, λ = 0.978; Neoaves: slope: − 0.041, R^2^ = 0.005, n = 4943, *p* < 0.001, λ = 0.871; Palaeognathae: slope: 0.125, R^2^ = 0.073, n = 36, *p* = 0.111, λ = 0.344). Clutch sizes are higher in Palaeognathae (mean 5.5) and, especially, in Galloanserae (mean 6.7) than in Neoaves (2.9 ± 1.2). In mammals, the relationship between litter size and body size is significantly negative, but very weak, in placentals (slope − 0.015, R^2^ = 0.002, n = 2272, *p* = 0.047, λ = 0.949), and marsupials (slope − 0.067, R^2^ = 0.044, n = 211, *p* = 0.002, λ = 0.994). In placentals, the relationship is decidedly non-linear, and an apparent decline mainly seems to show decreased variance in litter sizes as body sizes increase. Nonetheless, marsupials have larger litters than placentals (3.2 vs 2.5). Finally, in the Squamata as a whole (slope 0.201, R^2^ = 0.190, n = 3997, *p* < 0.001, λ = 0.902), in lizards (slope 0.186, R^2^ = 0.155, n = 3692, *p* < 0.001, λ = 0.908) and snakes (slope 0.260, R^2^ = 0.374, n = 305, *p* < 0.001, λ = 0.787) separately and, especially, in turtles (slope 0.399, R^2^ = 0.406, n = 283, *p* < 0.001, λ = 0.849) clutch size increases with body size (Fig. 6). The average brood size of turtles (12.1) is also much larger than the squamate equivalent (4.9 ± 5.3).

#### Reproductive frequency

**Fig. 7:**
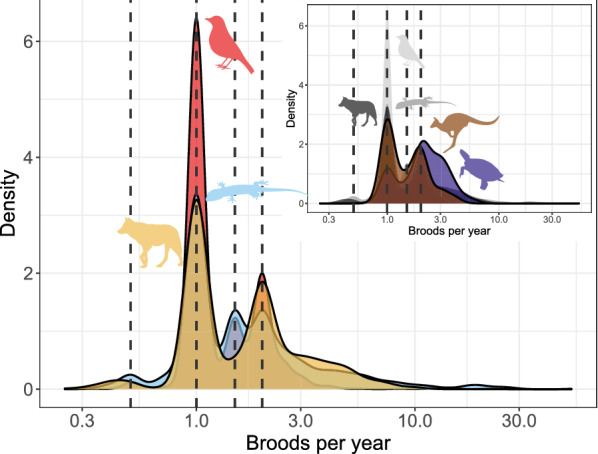
Broods per year. Red: birds; blue: squamates; purple: turtles; yellow: placental mammals; brown: marsupial mammals. Dashed vertical lines represent 0.5, 1, 1.5 and 2 broods per year. The x axis is log_10_ scaled

**Fig. 8:**
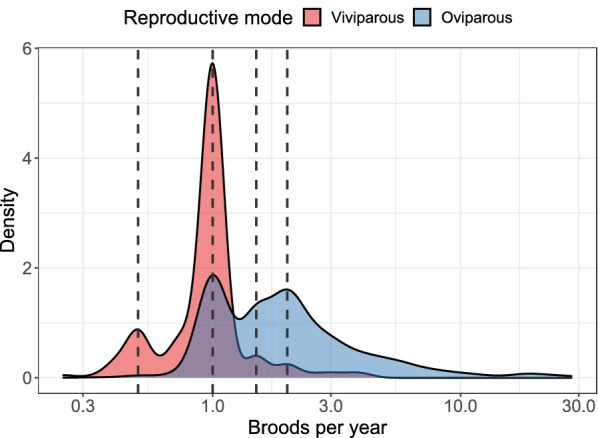
Parity mode and broods per year for the Squamata. Dashed vertical lines represent (from left to right) 0.5, 1, 1.5 and 2 broods per year. The x axis is in log_10_ scale

Ectotherms reproduce, on average, more frequently than endotherms. Turtles in our dataset (n = 182 species) reproduce, on average, 2.28 times a year, and squamates 2.22 times (n = 1584), while mammals (n = 889) reproduce 1.88 times, and birds 1.46 times a year (n = 1784). High values for squamates, however, mostly represent anole reproduction in captivity. Of the 25 species reproducing 15 or more times a year (range: 15–28), 24 are anoles (the other is the sphaerodactylid gecko, *Pristurus flavipunctatus* which produced 78 clutches in 43 months in captivity, [155]). All these 25 species produce 1 egg per clutch. Without these data the average for Squamata is 1.93—similar to the mammalian mean. The median number of broods per year is one for Aves, (Neoaves: 1, Galloanserae: 1, Palaeognathae: 1.5), 1.5 for Squamata (Sauria: 1, Serpentes: 1.5) and Mammalia (Eutheria: 1.5, Metatheria: 1.4) and 2 for Testudines. Two of three species reproducing once every four years are snakes (the Arafura file snake, *Acrochordus arafurae* and the Shedao Island pitviper, *Gloydius shedaoensis*); the third is the sperm whale (*Physeter catodon/macrocephalus*). The median reproductive frequency is lower in birds (1 clutch a year) than in mammals and squamates (1.5 broods a year) and is the highest in turtles (2). The mode, and overall shape of the distributions are, however, extremely similar across birds, mammals and squamates (Fig. 7). In these three taxa the mode is one clutch or litter per year (in turtles the mode is 3), with a second, lower mode at two, and a distinct group of species reproducing once or twice a year. The frequencies decrease as the number of reproductive events per year increases. Although the modal reproductive frequency was the same for oviparous and viviparous squamates (one brood per year), oviparous squamates had higher median and mean reproductive frequency (1.5 and 1.9, respectively), then viviparous species (1 and 0.9; Fig. 8), with a significant difference in the means when controlling for body mass and phylogeny (*p* = 0.019).

Within birds, Galloanserae lay a little more frequently (mean 1.67 clutches a year) than either Palaeognathae or Neoaves (1.43 in both). In mammals, placentals reproduce a little more frequently than marsupials (1.91 vs 1.60 litters year^−1^).

We highlight, however, that data on reproductive frequency is relatively scant. For example, the slowest reproducing species of amniotes are not in our database: the tuatara (*Sphenodon punctatus*) reproduces (almost) once every four years (e.g., [156]), while elephants reproduce even slower: Shoshani and Eisenberg [157] report that Asian elephants, *Elephas maximus*, reproduce once every 2.5–8 years, and Howard [158] reports 3–9 years for African elephants, *Loxodonta africana*. Data for elephant reproductive frequencies are absent from Jones et al. [104], and *Sphenodon* is not a squamate, therefore they were not analyzed here (Fig. 8).

#### Relative brood mass

**Fig. 9:**
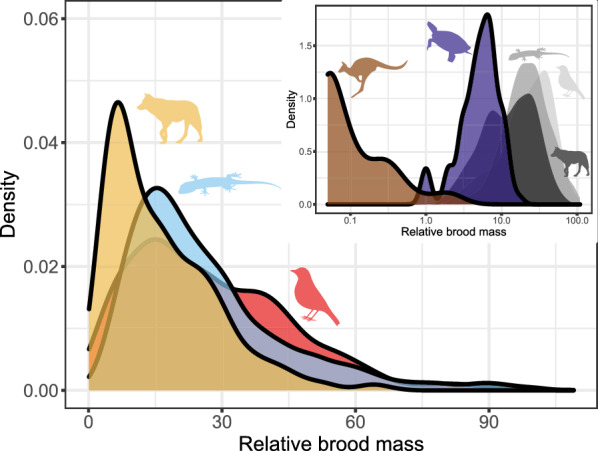
Relative brood mass. Red: birds; blue: squamates; purple: turtles; yellow: placental mammals; brown: marsupial mammals. Note that the x axis in the inset figure is log_10_ scaled

Overall, the three major taxa are very similar, showing a similar spread of values (though no mammal has a relative brood mass higher than 70%, whereas both birds and squamates have values close to 1, Fig. 9). In both squamates and birds the most common values are between 10 and 25%, but in eutherians lower values are also frequent, and the mode is 5–10%. Turtle clutches weigh relatively little, with 89% of the species in our dataset have broods weighing less than 10% of the female size (Fig. 9). In marsupials, the weight of the litter does not exceed 2.2% in any of the species for which we have data and is always much smaller than the other groups (Fig. 9, inset).

**Fig. 10:**
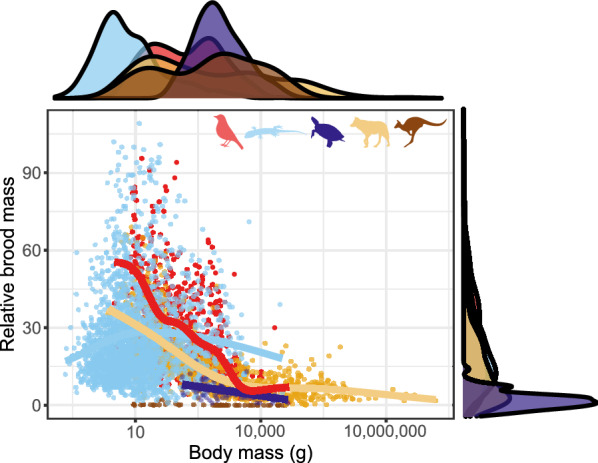
Allometry of relative brood mass (in % of female mass). Red: birds; blue: squamates; purple: turtles; yellow: placental mammals; brown: marsupial mammals. Note that density plot of relative brood mass and regression line for marsupials are not shown because values are very close to zero. Solid regression lines are from Generalized Additive Models with knots (K = 9). Note that x-axis is log_10_ scaled

Overall relative brood mass rapidly declines with increasing adult size in all taxa (Fig. 10). The weight of all offspring from a single brood, divided by female weight (‘relative brood mass’) varies greatly between less than 10% and nearly 100% of the size of the mother up to a maternal size of ~ 100 g in eutherians and ~ 1 kg in squamates and birds. Mammals have lower values at all sizes and grow to much larger sizes than both birds and squamates. Thus, the average value (0.163) is much lower in eutherians than in either squamates (0.265), or birds (0.275), which are very similar to each other. Turtles (0.059) and, especially, marsupials (0.002) have much lower values still (Fig. 10).

#### Yearly fecundity

**Fig. 11:**
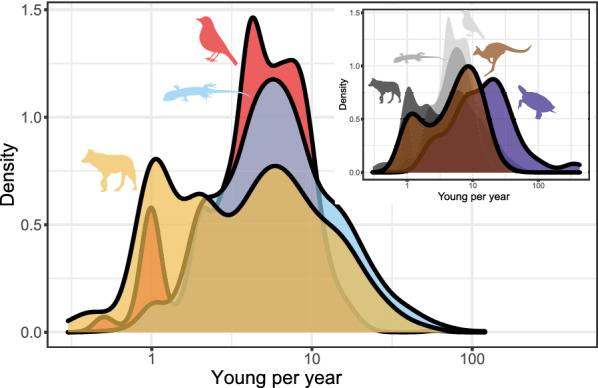
Young per year. Red: birds; blue: squamates; purple: turtles; yellow: placental mammals; brown: marsupial mammals. Note that the x axis is log_10_ scaled

The number of offspring per year is highly variable, ranging from one offspring per three years in some whales, and one per two years in some sea birds, large raptors, and the amphisbaenian *Blanus mettetali*—to over 50 in all three classes. Placentals (6.3 ± 7.2 neonates year^−1^, n = 787) and marsupials (6.9 ± 5.5 neonates year^−1^, n = 84) have similar yearly fecundity. In birds, Neoaves are much less fecund (4.9 ± 3.7 eggs year^−1^, n = 1514), than members of the Galloanserae (8.4 ± 3.3, n = 246) and Palaeognathae (9.4 ± 5.5, n = 16). Within reptiles, squamates (8.9 ± 9.5, n = 1557 species) are similar to the two latter bird groups, but turtles have by far the largest annual fecundities (31.1 ± 63.4, n = 181). Furthermore, while the mammalian mode is 1–2 offspring per year, and higher values are increasingly less frequent, squamates and birds have a right-skewed distribution with a mode of 4–6 young, and turtles have no distinct mode but are generally characterized by much higher values than any other clade (Fig. 11).

**Fig. 12:**
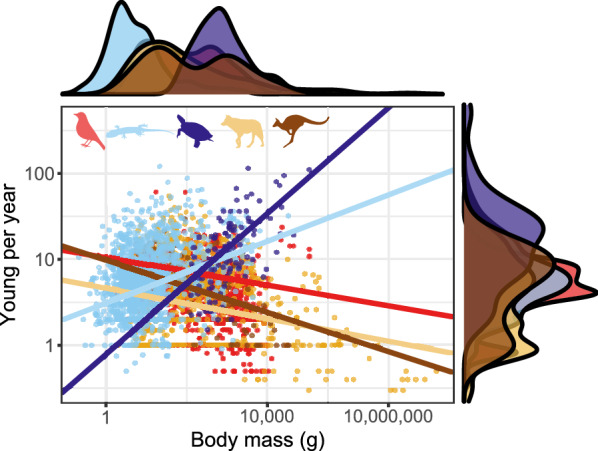
Allometry of young per year. Red: birds; blue: squamates; purple: turtles; yellow: placental mammals; brown: marsupial mammals. Solid lines are regression slopes from PGLS analyses. Note that both the axes are log_10_ scaled

In endotherms (birds, placentals and marsupials), larger-bodied species produce fewer offspring per unit time than smaller bodied ones, but larger ectotherms (turtles and squamates) out-produce smaller ones (Fig. 12).

#### Rates of reproductive output

**Fig. 13:**
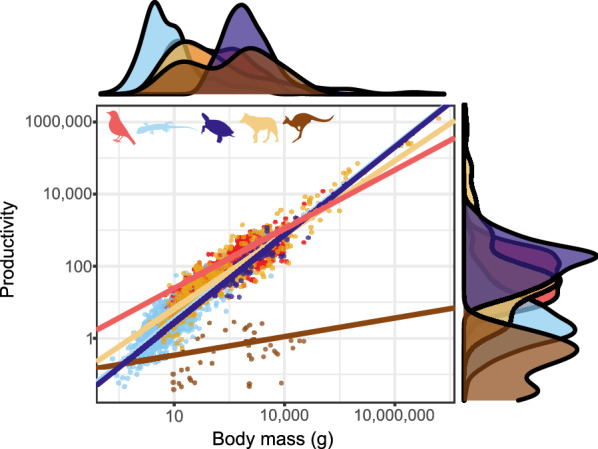
Allometry of yearly biomass productivity of amniotes. Red: birds; blue: squamates; purple: turtles; yellow: placental mammals; brown: marsupial mammals. Solid lines are regression slopes from PGLS analyses. Both axes are in log_10_ scale

Reproductive output rates, measured as the biomass of offspring hatching or being born in a year, are first and foremost tied to the size of the mother (Fig. 13). Compared across the amniote taxa the allometric slopes are somewhat dissimilar. They are shallowest in marsupials (slope: 0.179, R^2^ = 0.152, n = 43, λ = 0.941) followed by birds (slope: 0.549, R^2^ = 0.558, n = 978, λ = 0.893), steeper in placental mammals (slope: 0.689, R^2^ = 0.694, n = 467, λ = 0.805), and steeper still in turtles (slope: 0.816, R^2^ = 0.771, n = 75, λ = 0.142) and squamates (slope: 0.806, R^2^ = 0.633, n = 1053, λ = 0.753). The intercepts are also dissimilar: 0.602 in birds, 0.115 in placental mammals (*p* = 0.420), − 0.714 in marsupials, -0.396 in turtles, and -0.319 in squamates. These values, however, suggest more overlap than the differences imply, as shallow slopes are associated with high intercepts. Thus, the regression lines intersect at values close to 1 kg, where all classes are species-rich (Fig. 13). At somewhat smaller sizes, where most diversity resides in all taxa (except turtles), birds and mammals are somewhat more productive than squamates and, especially, turtles. That said, there is great overlap between all three major groups across the range of sizes where squamates, birds and mammals are most diverse (approximately 10–3000 g).

**Fig. 14:**
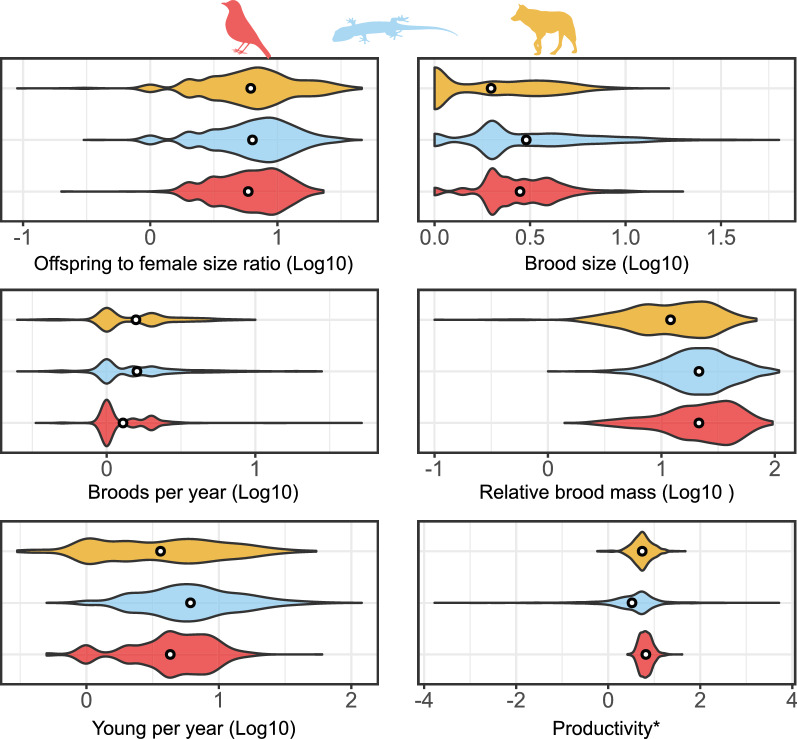
Violin plots depicting the distribution of life history traits in the three major amniote clades. Means are represented as solid white points. Productivity expressed as the ratio of productivity (brood size × brood frequency × offspring size) over adult body size to standardize for body size. Red: birds, blue: squamates, yellow: placental mammals. (See Additional file 2: Figure S1 for graphs that also include marsupials and turtles)

The overall patterns are quite similar across the three major amniote clades (Squamata, Aves and Eutheria) for most traits (Fig. 14), while members of the Metatheria and Testudines share similar values in some traits but differ markedly in others (Table 2).

**Table 2:** Summary table comparing life history distribution across amniotes

	Characteristics of distribution
Body mass	All unimodal and right skewed Squamata < Aves < < Mammals
Offspring/female size	Offspring/female size ratio approximately the same across placental mammals, birds and squamates (7–8%) marsupials < < < turtles < < birds ≈ placentals ≈ squamates
Brood size	Mode is 1 in mammals, 2 in birds and squamates
Reproductive frequency	The overall mode and distribution of reproductive frequency is extremely similar across amniote groups
Relative brood mass	All unimodal and right skewed. But no placental mammal has a relative brood mass higher than 70%. Small in turtles, very small in marsupials
Yearly fecundity	Distribution of reptiles and birds very similar (right skewed distribution with a mode of 4–6 young). Mammalian mode is 1–2 offspring per year
Reproductive output	Similar overall in squamates, birds and mammals (low intercepts associated with steep slopes), low intercept in turtles, very low intercept, and shallow slope in marsupials

## Discussion

Amniotes are distinct from anamniote vertebrates in their physiology and reproduction physiology. The evolution of the cleidoic egg has put them on a path that enables successful reproduction on land. It allows the embryos to develop, grow, and exchange gasses with the environment without extensive risk of desiccation. The evolution of the extraembryonic membranes of amniotes may be imposing a constraint on the lower limit of amniote egg—or embryo sizes [12, 88]. This constraint is probably absent in our anamniote kin and thus anamniotes’ eggs are often much smaller, even in large bodied species.

Amniotes then diversified into endotherms (twice), shelled ectotherms (once), and a major lepidosaur/squamate radiation that retained the primitive position of ectothermy without a shell. Endotherms evolved complex and prolonged thermal and food provisioning for their young, and often complex social systems, that reptiles mostly did not. Viviparity evolved multiple times, and within mammals takes two very distinct forms. All this, and more, certainly influenced the reproductive and life history characteristics of amniote taxa. Surprisingly, however, our hypotheses, that these major transitions will manifest in substantial quantitative differences between clades, were mostly refuted.

The evolution of endothermy and its abovementioned associated life-history traits, were unique evolutionary events (as is the evolution of the turtle shell). Claiming that endothermy is the cause of parental provisioning, or of inter-clade differences in the traits we study, is risky [99]. While associating the evolution of parental provisioning with endothermy is compelling [31], it is difficult to pinpoint endothermy as the cause of life history discrepancies without testing the myriad of other traits that evolved in mammals, birds, squamates, or turtles, during the hundreds of millions of years of their independent evolution. Furthermore, one is faced with a difficulty when analyzing the effects of such unique events quantitatively [146]. Because the species within each clade are not evolutionary independent units, the number of degrees of freedom for statistical analyses will be inflated if we treat each species level datum as an independent statistical entity. Phylogenetic comparative methods, on the other hand, are shackled by the few transitions to endothermy, and the non-independence of provisioning from endothermy. This may make them overly conservative. We therefore opted not to formally compare these trends across mammals, birds, squamates and turtles, but rather to view their overall patterns. We do not doubt that there are inter-clade differences in most of the traits we examined, and that many differences that will be deemed ‘statistically significant’, and will result in models with relatively low AIC values, etc. We contend, however, that the overall picture is of great similarity across the major amniote taxa (Aves, Theria, Squamata) in most of the traits we examined.

Offspring sizes increase linearly with adult (or female) masses in placentals, birds and squamates, and the offspring are generally of similar size relative to that of the mother (Fig. 4). Brood sizes have similar modes and distributions, with the caveats that a litter of one is the mode in mammals, but two in birds and squamates—and very large broods are much more common in squamates and, especially, turtles. Most taxa in all clades reproduce once or twice a year (Fig. 5). Again, some squamate groups (usually with a clutch of one or two eggs, e.g., anoles) are ‘outliers’. But most taxa share very similar values across classes. We suspect that similar frequencies may characterize other vertebrate and potentially invertebrate taxa as well.

Bird and squamate clutches are also very similar in terms of their mass relative to that of the mother (Fig. 9). This is despite the avian eggs losing water during incubation [159] while the eggs of most squamates take up water from the environment ([160]; though some reptile clades, such as most gekkotans, are more bird-like in this respect). This is even more surprising given that birds develop their eggs sequentially within a single clutch while squamate eggs belonging to the same clutch develop simultaneously within the mother’s reproductive tract [161]. Placental litter masses are smaller, both relative to the size of the adults, and because of the abundance of very large mammals coupled with the negative relationship between body size and relative litter mass. An additional reason may be the extra weight of the complex placenta and associated tissues. That said, viviparous and oviparous squamates do not differ in this respect [72]. Yearly fecundity shows much variation across taxa. Though birds and squamates are quite similar overall, there are large differences between major avian clades, and between major squamate clades.

Given the overall similarities, the fact that biomass of offspring produced by a female in a year is so similar across clades, when viewed in term of its relationship with the mass of the females (especially across a similar range of female masses), could be expected. It seems as if the major differences in reproductive physiology between these taxa and hundreds of millions of years of subsequent independent evolution, had surprisingly little effect on the quantitative traits that we examined (Fig. 13; see also [162]). While the paths may differ greatly, some fundamental constraints impose overall similar outcomes, despite very different ‘solutions’ and adaptations.

Two deeply divergent lineages, however, depart much from their relatives. Turtles differ both from birds (their sister clade) and from squamates (while sharing their ectothermic physiology). Turtles reproduce frequently, which is even more remarkable given their large size (and perceived overall slowness). They also lay many small eggs per clutch. Despite this, they have low relative clutch masses: the large clutch size does not fully compensate for the small size of the hatchlings. Thus, turtles also have low rates of biomass production. Turtles have a very long independent evolutionary history, and thus differ from other amniotes in many traits. It is nonetheless tempting to advocate that the life history discrepancy we identify between turtles and other amniotes is caused by the hallmark of turtle-ness, their shell. We hypothesize that the evolution of the rigid shell imposes an upper limit on egg size. This is of course not a new hypothesis (one can find it in textbooks, e.g., [68]), but we only found it studied within turtle species, and across small groups of closely related species, that do not span the size range of turtles as a whole (e.g., [163]). Interestingly, both Congdon and Gibbons [163], and Rollinson and Brooks [164], identified such constraints to be stronger in small species, and small females, respectively, whereas the shallow egg size-body size allometric slope we obtained suggest that larger species may be more strongly constrained. Janzen and Warner [165] found that offspring fitness was maximized at larger egg size than the size which maximized maternal fitness. Furthermore, they found that actual egg sizes were closer to the maternal than to the offspring optimum, suggesting that selection on turtle egg size was driven by selection on the females rather than on their offspring [165]. Our results agree with this suggestion. We hypothesize that the constraint on egg sizes in turtles has strong downstream implications for the evolution of clutch size and clutch frequency. Beyond the external shell of tortoises, greater egg production may be limited by calcium requirements [166–168]. Calcium is needed for skeletal development and maintenance, and for egg-shell production [169]. Calcium requirements for egg production is higher for when many small eggs than for a few large ones of the same volume. Therefore, both limits on egg size and their numbers could potentially explain the unique pattern we identify in turtle reproduction (which may be more similar to what is found in some anamniote taxa) compared with other amniotes.

Marsupial reproductive traits are even more distinct than those of turtles. The tiny offspring of metatherians are much smaller than those of all other amniotes, regardless of their adult size. Interestingly, this does not seem to allow marsupials to have large litters, and indeed the decline in litter size with body size is stronger in marsupials than in placentals. Nor do metatherians reproduce more frequently—if anything they reproduce less often than placentals. Thus, their clutch masses, and annual biomass production, are much lower than in any of the other groups. If any groups of amniotes evolved a completely unique set of life history characteristics it is most probably marsupials. The huge discrepancy between the reproduction of marsupials and other amniotes may mean that some of the comparisons we made do not strictly contrast like with like. While the traits we chose are similar and easy to quantify across taxa, the extra ‘gestation’ of metatherian neonates in the mother’s pouch makes them physiologically and ecologically different to those of other taxa. Furthermore, the provisioning of young by mammals, and most birds, in the first stages of their lives may also mean that a more valid comparison with reptile offspring should be made at the weaning or fledgling stages. This however necessitates quantifying the rates of survival from birth and laying to weaning and fledgling and may also not be strictly comparable across taxa.

One way or another, marsupials differ much from placentals, and from all other amniotes, in the size of their neonates. Hamilton et al. [102], however, have shown that in terms of weaning mass, lifespan and, at least for large species, the time since conception to weaning and age at first reproduction, marsupials and placentals are remarkably similar. Thus, the very different way marsupials go about their reproduction again results in great similarities between them and placental mammals, if not other amniotes (or even vertebrates) in general.

Similar to the marsupial/placental contrast, deep splits in the avian and squamate trees of life do result in differences in traits such as those we studied here. For example, palaeognaths are more similar to reptiles (and much of the animal world) in having a positive clutch size/body size allometry, whereas neognaths have a basically flat (or slightly negative) allometry. This suggests that the forces affecting the relation between body size and clutch size evolved after the Palaeognathae/Neognathae split. However, members of both the Palaeognathae and Galloanserae lay more eggs than Neoaves birds, despite the Galloanserae being more closely related to the Neoaves.

Invariably there would be further differences between subclades the lower we go in the phylogenetic hierarchy. We did not attempt to quantify data for crocodiles or monotremes, because such small groups do not lend themselves easily to the type of generalizations we aimed to make. Nonetheless, differences in reproductive characteristics within major clades are well known. These include, for example, the small litter sizes and long lifespans of bats relative to other mammals [170, 171], and the fixed small clutches, and frequent laying of anoles and geckos [99, 133]. Overall, though, endothermy, and parental provisioning, do not seem to impose great constraints on traits such as brood size, offspring size, reproduction frequency, and their derivatives such as annual fecundity, relative brood mass and annual biomass production. This is reflected both in the similar ranges, modal values, and similar distributions of the traits we analyze. We are therefore more impressed with the overall similarities, than with the differences across taxa.

What then does explain the huge variation, observed in all major taxa, across the life history traits we examined? Body size is an obvious candidate and has long been known to be associated with life history traits (e.g., [76, 77]). We suspect that geography, manifested via climate and differences in species richness and guild composition (e.g., via insularity), are additional factors that affect vertebrate reproduction and aging. The effects of climate on life history traits have been a subject of extensive research, especially in birds (e.g., [172–175], see also Iverson [176] for turtles and Meiri et al. [58, 97] for lizards). One can wonder, for example, whether marsupials grow slowly [102], and reproduce less frequently (see above), than placentals because of their different reproductive physiology—or because most species (especially most species for which data are available) reside in Australia. Australia has relatively few mammal species (e.g., [177]), but an overabundance of lizards which is often thought to be a consequence of the nutrient poor conditions in much of the continents [178–182]. Yom-Tov [113] has shown that Australian passerines, especially from lineages that have colonized the continent early (‘old endemics’), reproduce more slowly than N. American passerines. A comparison between marsupials and placentals in Australia and South America is an obvious test of this hypothesis but is beyond the scope of this work.

We suspect that seasonality constrains the length of vertebrate activity season, influencing reproductive patterns. In more seasonal climates one option is to delay maturation, and potentially hibernate during the less suitable season. Alternatively, development may accelerate so animals are reproductively active by the onset of winter—or when they emerge in spring. Birds, for example, follow the second option, fledging early at high latitudes in very seasonal climates [119, 183].

Vertebrate zoologists often identify themselves as herpetologists, ichthyologists, ornithologists, or mammologists. Therefore, studies within classes are common, whereas cross-taxon, especially cross-class studies are rare (but see e.g., [90, 121, 162, 184, 185]). In biogeography and conservation, however, studies of terrestrial vertebrates are relatively common, as maps for all four classes and conservation statuses for (nearly) all amphibians, birds and mammals are thought to be available (though completeness varies). In terms of traits, however, the scarcity of cross-taxon studies therefore means we often have little feel for the similarities—or differences across taxa. We hope we have shown here that there is little reason why this should be the case. For us, the similarities were much more impressive than the differences that the fundamental divisions of physiology, locomotion, parental care, and sociality suggested would be the case. We hypothesize that the evolution of the cleidoic egg was the most influential step setting amniotes apart from anamniotes. Whether the differences between these two groups stretches to more than differences in clutch and offspring sizes remains to be explored. We hope studies across amniote, tetrapod, and vertebrate clades (and even across vertebrates and invertebrates) will become much more common.

## Supplementary Information

**Additional file 1: Appendix S1.** Amniote body sizes.

**Additional file 2: Figure S1.** Reproductive characteristics of birds, squamates, turtles, placentals, marsupials and turtles.

## Data Availability

All data used in this paper appear in Additional file 1: Appendix S1, or were already published (and full citations are provided).
