# Supplementary material for: Different solutions lead to similar life history traits across the great divides of the amniote tree of life
Source: J Biol Res (Thessalon). 2021 Feb 8;28:3. doi: 10.1186/s40709-021-00134-9 (PMC7869468; doi:10.1186/s40709-021-00134-9)
Supplement: Supplementary file 2 — Additional file 2: Figure S1. Reproductive characteristics of birds, squamates, turtles, placentals, marsupials and turtles. [file 40709_2021_134_MOESM2_ESM.pdf]

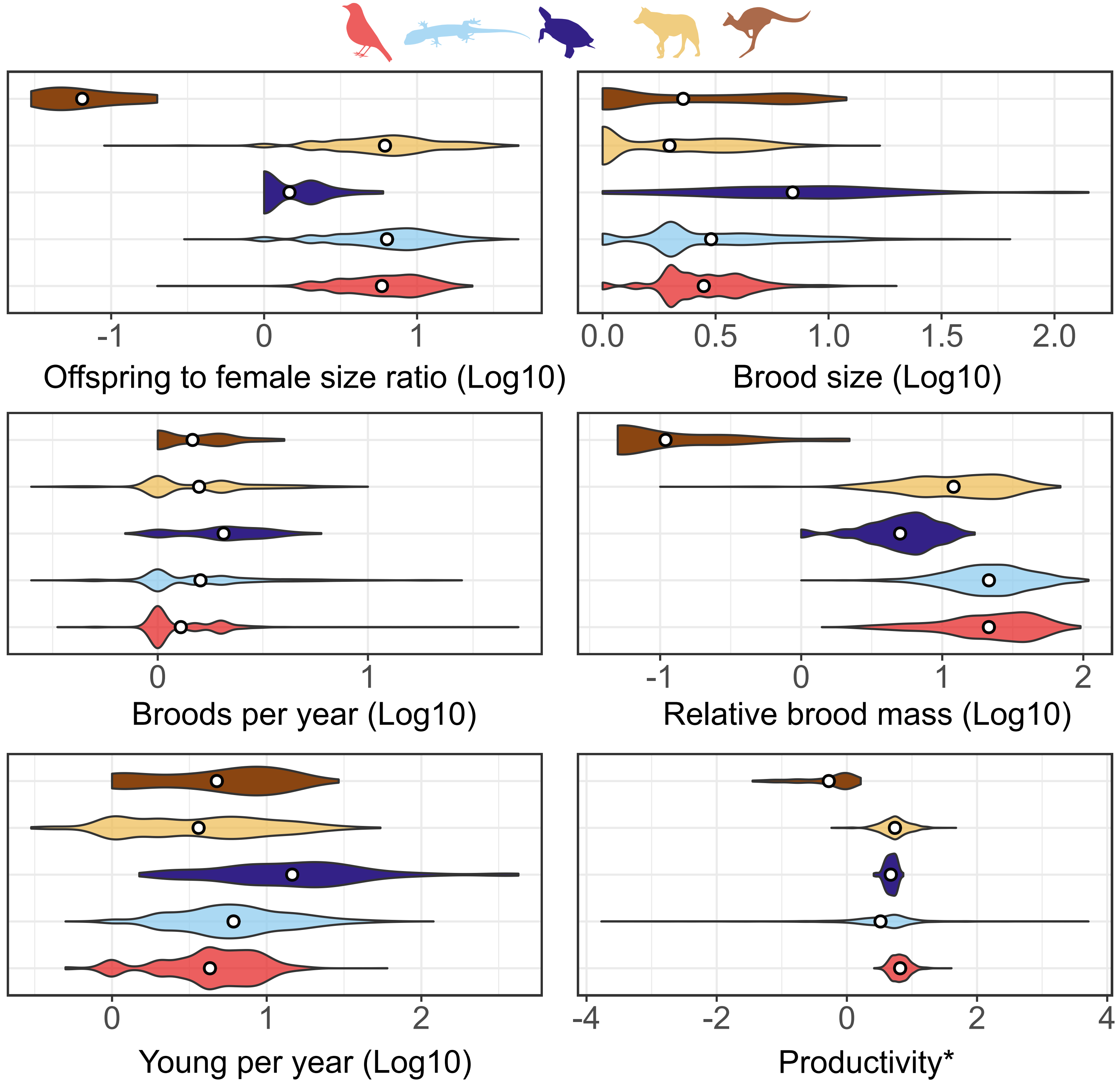
-1
0
1
Offspring to female size ratio (Log10)
0.0
0.5
1.0
1.5
2.0
Brood size (Log10)
0
1
Broods per year (Log10)
-1
0
1
2
Relative brood mass (Log10)
0
1
2
Young per year (Log10)
-4
-2
0
2
4
Productivity*
